# Causal effects of education attainment on oral and oropharyngeal cancer: New evidence from a meta-analysis and Mendelian randomization study

**DOI:** 10.3389/fpubh.2023.1132035

**Published:** 2023-04-12

**Authors:** Gui Chen, Junyang Xie, Di Liu, Xiaowen Zhang, Anzhou Tang

**Affiliations:** ^1^Department of Otolaryngology Head and Neck Surgery, The First Affiliated Hospital of Guangxi Medical University, Nanning, China; ^2^State Key Laboratory of Respiratory Disease, Department of Otolaryngology-Head and Neck Surgery, The First Affiliated Hospital of Guangzhou Medical University, Guangzhou, Guangdong, China

**Keywords:** meta-analysis, Mendelian randomization, oral cavity and pharyngeal cancer, association, education attainment, genome-wide association study

## Abstract

**Objectives:**

Higher educational attainment (EA) has proven to be beneficial for preventing and treating various types of cancers. Currently, there is little evidence on the association between EA and prevention of oral cavity and pharyngeal cancer (OCPC).

**Methods:**

Several databases were searched until October 1, 2022, and a meta-analysis was performed. A Mendelian randomization (MR) study was conducted with EA (i.e., the exposure) data derived from the Social Science Genetic Association Consortium and 6,034 cases of OCPC (i.e., outcome) selected from the Integrative Epidemiology Unit genome-wide association study. Five methods were used to evaluate the causality between EA and OCPC. The leave-one-out sensitivity test, MR-Egger regression, and multivariable MR (MVMR) analysis were applied to evaluate the MR results.

**Results:**

The meta-analysis included 36 eligible studies. EA was significantly and negatively associated with OCPC risk (odds ratio [OR]: 0.439, 95% confidence interval [CI]: 0.383–0.503, *P* < 0.001). MR analysis revealed that the risk of OCPC, oropharyngeal cancer, and oral cavity cancer decreased with an increase in education (OR: 0.349, 95% CI: 0.222–0.548, *P* < 0.001; OR: 0.343, 95% CI: 0.198–0.597; *P* < 0.001; OR: 0.342, 95% CI: 0.195–0.601, *P* < 0.001, respectively). Even after correcting for mediators, high EA still significantly reduced the risk of OCPC (OR: 0.361, 95% CI: 0.281–0.463, *P* < 0.001).

**Conclusion:**

Both the meta-analysis and MR results demonstrated that high levels of EA can reduce the risk of OCPC in the general population.

## 1. Introduction

The prevalence of oral and oropharyngeal cancer (OCPC) has increased in recent years, making it the sixth most frequent neoplasm (1, 2). Due to the limited efficacy of current treatment methods, the 5-year survival rate of patients with OCPC is reported to be ~50% in Europe, and the survival rate is expected to be even lower in developing countries (3). The principal risk factors of OCPC include smoking, alcohol abuse, and age older than 40 years. In addition, human papillomavirus (HPV) infection is an important risk factor for oropharyngeal cancer (OPC). These factors have been incorporated into disease prevention strategies (4, 5). Early identification of potentially modifiable risk factors and incorporation into prevention strategies may help prevent the occurrence and development of OCPC (6). Numerous studies have shown that high educational attainment (EA) is highly beneficial in chronic disease and cancer management; therefor, EA can be used as an important prevention strategy (7, 8). Similarly, high EA may be a protective factor against OCPC; in previous studies, high EA was related to a reduction in the risk of this OCPC (9–11). However, observational research has several methodological limitations that make it difficult to clarify the association between EA and OCPC. Furthermore, conducting randomized controlled trials in this field is an unrealistic endeavor. Recently, Mendelian randomization (MR) has emerged as a new epidemiological tool to assess causality based on genetic variations associated with random exposure (12). The MR method has successfully identified causal relationships between EA and several diseases (13–15). This study aimed to conduct an updated meta-analysis and MR study to evaluate the relationship between EA and OCPC. Our results may be used as robust evidence for formulating preventive policies.

## 2. Materials and methods

### 2.1. Search strategy in meta-analysis

This meta-analysis was registered in PROSPERO (ID: CRD4202365834). Databases including PubMed, Web of Science, Embase, and Cochrane Library were searched until October 1, 2022. The searches were conducted using the following medical subject heading (MESH) terms: “education,” “oral cavity,” and “pharyngeal cancer;” these terms and their variants were linked using Boolean operators.

### 2.2. Study selection

Studies meeting the following criteria were included: (1) focus on education and OCPC (including broader definitions such as head and neck cancer) that reported odds ratios (ORs), with 95% confidence intervals (95% CIs), or adequate data to calculate these measures, (2) inclusion of participants aged 18 years or older, (3) availability of the full text of the study and published in English, and (4) sample size of ≥200. Review articles, meta-analyses, case reports, and editorials were excluded. Two researchers (GC and JX) independently assessed the full texts after screening all the titles and abstracts.

### 2.3. Data extraction and quality assessment

Data on the full title of a study, first author, year of publication, study design, follow-up period, sources of cases and participants, number of cases and controls, and ORs were independently extracted by two investigators (GC and JX). Since the studies included education levels assessed using different methods and scales, we redefined education level to facilitate data collection and comparison. Therefore, for each study, we defined the lowest category reported in the study as the lower education level and the highest category as the higher education level. The Newcastle Ottawa Scale (NOS) tool was used to evaluate the quality of each study (low, medium, and high quality). Any disagreements between the researchers were resolved through consensus.

### 2.4. Statistical analysis of meta-analysis

Statistical analyses were conducted using Stata 14.0. *I*^2^ analysis was used to assess heterogeneity, and an *I*^2^-value >50% indicated heterogeneity between the studies. Data synthesis based on different research populations and methods was conducted using a random-effects model. Subgroup and meta-regression analyses were performed. The robustness of the literature results was evaluated by sensitivity analysis. Funnel plots derived from the Egger and Berger bias tests were used to check for publication bias.

### 2.5. Dataset of exposures

A genome-wide association study (GWAS) dataset, which included 766,345 individuals of European descent who had received an education, was selected as the exposure set (Supplementary Table 1) (16). These individuals were aged older than 30 years, and EA was measured as the number of completed schooling years. Years of schooling were converted and standardized, where each unit represented 4.2 years of schooling (1 standard deviation [SD] = 4.2 years). Based on published MR studies (17, 18), a total of 268 single nucleotide polymorphisms (SNPs) were selected in our study for MR analysis (Supplementary Table 2). According to previous studies, these SNPs accounted for 12% of the variation in EA among individuals (16). The tools used in these studies can be considered to have a strong predictive effect on education level since the F-statistic was 41.76 (19).

### 2.6. Dataset of outcomes

The Integrative Epidemiology Unit GWAS database, which included 6,034 cases of OCPC and 6,585 controls, was selected as the outcome set (Supplementary Table 1) (20). The cases included were in line with the International Classification of Diseases 10 (ICD-10) standard. We focused on participants of European descent, including patients with OCPC, oral cavity cancer (OCC), and OPC.

### 2.7. EA and common risk factors for OCPC

To explore the potential mediators of the EA–OCPC pathway, we used the inverse-variance weighted (IVW) method to assess the potential causal relationship between EA and common risk factors for OCPC. Based on the current database, we analyzed the following common risk factors of OCPC, including HPV, number of sexual partners in lifetime, hypertension, type 2 diabetes, smoking, and alcohol consumption. Supplementary Table 1 presents the GWAS summary data of the above risk factors. Multivariable MR (MVMR) was mainly used to evaluate the impact of multiple potential exposures on the results as a whole and to identify potential risk factors.

### 2.8. Statistical analysis of MR

According to the guidelines for MR, several MR approaches, including the IVW method, weighted median method, MR-Egger method, simple mode, and weighted mode, were used to estimate the relationship between EA and OCPC. The IVW method is primarily used to evaluate causal relationships since it possesses the highest statistical power. ORs were transformed with effect estimates (equivalent to beta coefficients), and the results were represented with 95% CIs. Since many instrumental variables (IVs) were associated with multiple traits (pleiotropy), it was necessary to apply sensitivity analysis to the results. Therefore, we applied MR-Egger regression and the leave-one-out sensitivity test. If the regression intercept was close to 0, it indicated the absence of horizontal pleiotropy (21). The leave-one-out sensitivity test was mainly used to calculate the MR results of the remaining IVs after removing the IVs one by one. No difference between the estimated MR result and the result after removing an IV indicated that the MR result was robust. All MR analyses in this study were conducted using the TwoSampleMR package in R (22).

## 3. Results

### 3.1. Article selection and quality assessment

**Figure 1:**
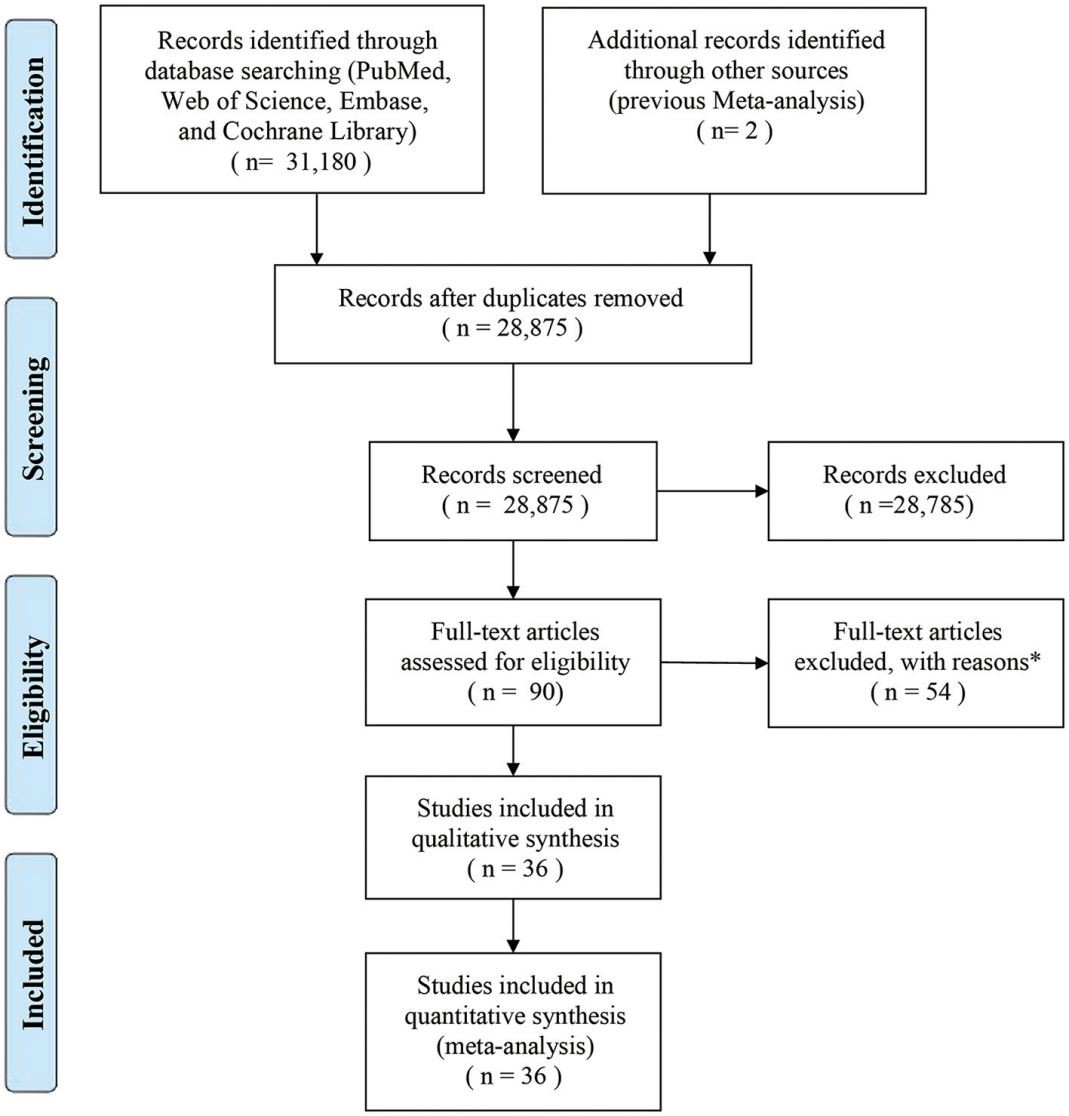
Flow diagram of the search strategy and identification of studies used in the meta-analysis. *Reasons for the exclusion of 54 studies were as follows: 38 studies lacked sufficient data, 15 studies had small sample sizes, and one study was not published in English.

A total of 36 studies on OCPC (including 13 studies that delineated their focus on OCC) were finally included in the meta-analysis (Figure 1). All included studies had a case-control design, and their characteristics have been summarized (Supplementary Tables 3, 4). In terms of geographical location, six studies were conducted in Europe, 21 studies in the Americas, and nine in Asia. There were 67,326 cases (OCPC) and 37,903 controls (non-OCPC) in the 36 studies. NOS assessment revealed a moderate risk of bias.

### 3.2. Meta-analysis

**Figure 2:**
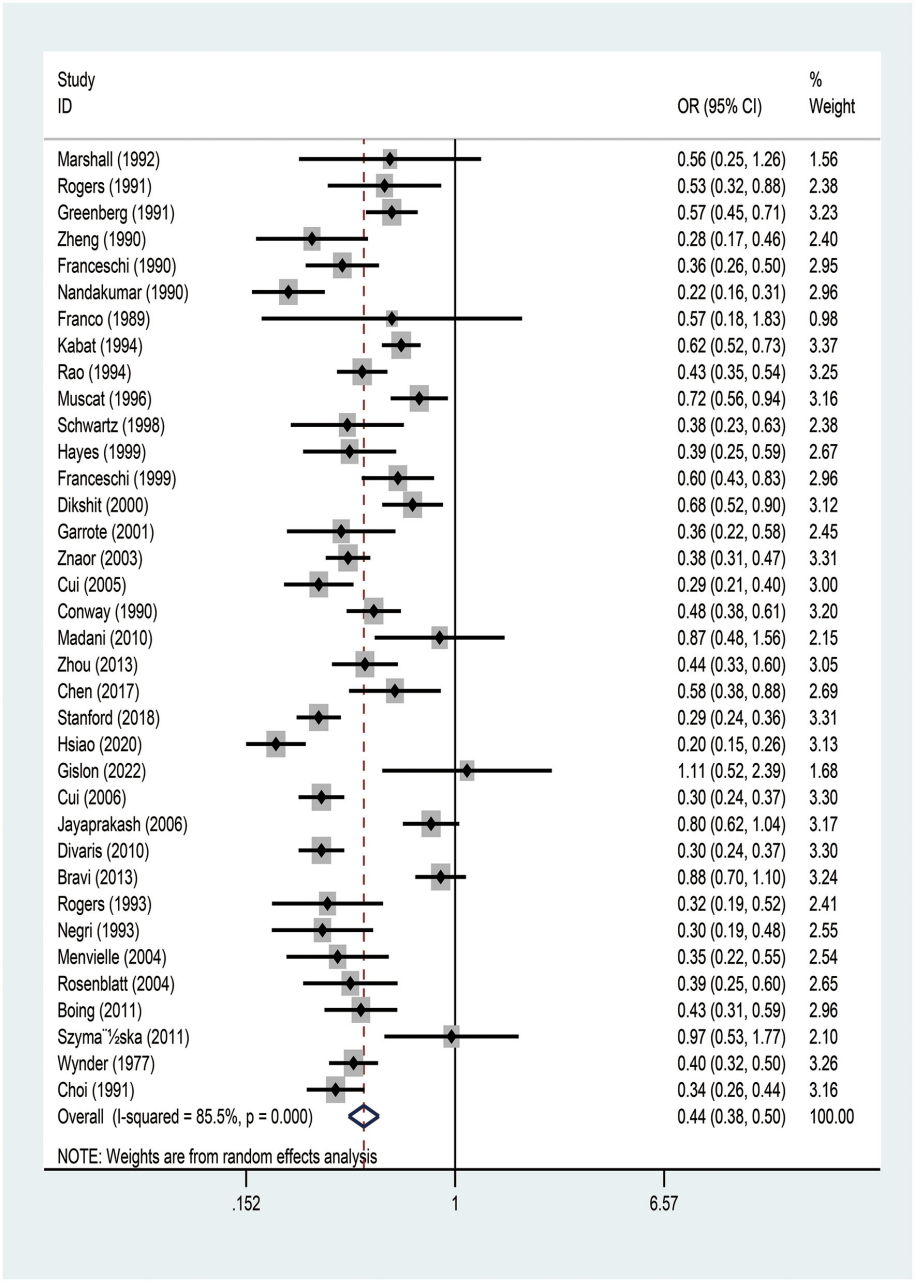
Meta-analysis of studies on education and the risk of oral and oropharyngeal cancer using the random-effects model ordered by date of publication. OR, odds ratio; CI, confidence interval.

The meta-analysis of the studies on the relationship between EA and OCPC revealed significant heterogeneity (*I*^2^ = 92.7%, *P* < 0.001). Random-effects model analysis showed a significant negative association between EA and OCPC, and the pooled OR was 0.439 (95% CI: 0.383–0.503, *P* < 0.001). The forest plots showed 36 studies (Figure 2). In the subgroup analysis, no heterogeneity was found in region, quality, year of publication, or sample size (Supplementary Table 5). The meta-regression analysis showed no significant heterogeneity in the year of publication, sample size, and quality score (Supplementary Table 6). The combined results of the remaining studies did not change significantly after the sequential elimination of each study in sensitivity analysis. The funnel plots, Egger's test results (*P* = 0.775), and Berger's test results (*P* = 0.505) confirmed that there was no publication bias (Supplementary Figure 1). We further explored the relationship between education and OCC (including 13 studies that clearly defined OCC): our random-effects model analysis showed a significant negative association between EA and OCC (pooled OR = 0.425, 95% CI: 0.345–0.549; *P* < 0.001) (Supplementary Figure 2). Subgroup analysis indicated that the existing heterogeneity may have been caused by region and quality (Supplementary Table 7). Further meta-regression analysis showed no significant heterogeneity in year of publication, sample size, or quality (Supplementary Table 8). After the sequential elimination of each study, the combined results of the remaining studies did not change significantly. The funnel plot, Egger's test results (*P* = 0.913), and Berger's test results (*P* = 0.054) showed no evidence of publication bias.

### 3.3. MR analysis

There was a significant causal effect of EA on OCPC in the IVW method (OR: 0.349, 95% CI: 0.222–0.548, *P* < 0.001). Additionally, a positive result was obtained by applying the weighted median method (OR: 0.303, 95% CI: 0.155–0.592, *P* < 0.001). Although no statistical significance was found, similar results were found with the MR-Egger, simple mode, and weighted mode methods (Table 1). The slope of the MR scatter diagram represents the impact of the exposure on the outcome. The MR fitting results showed that risks of OCPC, OCC, and OPC decreased with the increase in EA (Supplementary Figure 3). Since the intercept in MR-Egger regression was close to 0 (intercept β = 0.003; SE = 0.012, *P* = 0.800), it was unlikely to have horizontal pleiotropy (Supplementary Table 9). In addition, the forest plot showed that the MR fitting line was completely on the left side of 0, indicating that the increase in EA level reduced the risk of OCPC (Supplementary Figure 4). The funnel plots in this study were symmetrical; therefore, no pleiotropic effects were observed (Supplementary Figure 5). Sensitivity analysis yielded robust evidence, and the results were not affected by any SNP (Supplementary Figure 6).

**Table 1:** MR estimates of the causal effect of EA on OCPC, OCC, and OPC.

**Models**	**OCPC**		**OPC**		**OCC**	
	**OR (95% CI)**	***P*-value**	**OR (95% CI)**	***P*-value**	**OR (95% CI)**	***P*-value**
MR Egger	0.280 (0.048–1.640)	0.159	0.180 (0.021–1.561)	0.121	0.432 (0.047–3.945)	0.458
Weighted median	0.303 (0.155–0.592)	< 0.001	0.452 (0.197–1.037)	0.061	0.260 (0.115–0.588)	0.001
Inverse variance weighted	0.349 (0.222–0.548)	< 0.001	0.343 (0.198–0.597)	< 0.001	0.342 (0.195–0.601)	< 0.001
Simple mode	0.237 (0.025–2.264)	0.212	0.442 (0.027–7.301)	0.569	0.299 (0.019–4.536)	0.385
Weighted mode	0.237 (0.042–1.333)	0.103	0.813 (0.078–8.517)	0.863	0.219 (0.029–1.684)	0.146

MR, Mendelian randomization; EA, educational attainment; OCPC, oral cavity and pharyngeal cancer; OPC, oropharyngeal cancer; OCC, oral cavity cancer; OR, odds ratio; CI, confidence interval.

Further exploration of the relationship between EA, OPC, and OCC showed that EA was significantly associated with OPC and OCC (Table 1). MR-Egger regression showed the absence of horizontal pleiotropy (Supplementary Table 9). Similarly, the funnel plot and leave-one-out sensitivity analysis showed that MR results were reliable.

Assessing the causal relationship between EA and the common risk factors of OCPC was conducive to exploring the interference factors that may mediate the association between EA and OCPC. The preliminary results showed that 4.2 years of additional education was significantly related to a reduced risk of common risk factors (including BMI, hypertension, type 2 diabetes, and smoking) (Table 2). To control the pleiotropic pathway, when we further applied the MVMR model after correcting for the above mediating factors, education level still had a protective effect against OCPC (OR: 0.361, 95% CI: 0.281–0.463, *P* < 0.001) (Table 3).

**Table 2:** Causal effects of EA on common risk factors of OCPC according to IVW method.

**Outcomes**	**SNPs, *n***	**OCPC**	
		**OR (95% CI)**	**MR** ***p*****-value**
HPV E7 Type 16	28	1.494 (0.311–7.176)	0.616
HPV E7 Type 18	28	3.496 (0.668–18.285)	0.138
Number of sexual partners in lifetime	299	0.997 (0.965–1.030)	0.849
Hypertension	300	0.997 (0.996 −0.998)	< 0.001
Type 2 diabetes	297	0.676 (0.563–0.811)	< 0.001
Cigarettes per day	294	0.702 (0.650–0.758)	< 0.001
Alcoholic drinks per week	293	1.042 (1.011–1.074)	0.008

EA, educational attainment; OCPC, oral cavity and pharyngeal cancer; n, number; SNP, single-nucleotide polymorphism; OR, odds ratio; CI, confidence interval; HPV, human papillomavirus infection; IVW, inverse-variance weighted.

**Table 3:** MVMR results for OCPC.

**Outcome**	**Exposure**	**SNPs, *n***	**OR (95% CI)**	***P*-value**
OCPC	EA	277	0.361 (0.281–0.463)	< 0.001
	Hypertension	0	NA	NA
	Type 2 diabetes	7	1.000 (0.922–1.085)	0.996
	Cigarettes per day	8	1.334 (1.102–1.613)	0.131
	Alcoholic drinks per week	16	8.864 (4.934–15.924)	0.002

MVMR, multivariable Mendelian randomization; n, number; SNP, single-nucleotide polymorphism; EA, educational attainment; OCPC, oral cavity and pharyngeal cancer.

## 4. Discussion

In our study, we found that EA was negatively associated with the risks of OCPC, OPC, and OCC. Specifically, according to MR, 4.2 years of additional education lowered the risk of OCPC, OPC, and OCC by 65.1%, 65.7%, and 65.8%, respectively. These results revealed a cause-and-effect relationship between high EA and low risk of OCPC.

Cancer prevention has long been considered one of the most effective strategies to overcome this public health problem, and some experts consider certain cancers to be major but preventable chronic life-threatening diseases (23). Therefore, educational programs to raise public awareness of the risk factors for cancer and promote healthy lifestyles are important measures for primary cancer prevention (24). Education is inversely associated with the incidence of several types of cancer. In other words, the more educated an individual is, the lower the risk of developing cancer. Observational studies have reported that socioeconomic status (including education) is closely associated with the risk of head and neck cancer. A study showed that the incidence of OCPC in uneducated populations is significantly higher than that in educated populations (9).

A previous meta-analysis showed that higher education level was protective against OCPC (25, 26). However, it should be noted that most of the studies included in the meta-analysis had a case-control design with a small sample size. In contrast, we only included studies with more than 200 cases and also included recent studies to enhance the reliability of our results. Although our meta-analysis showed that education was a protective factor for OCPC, it was difficult to determine a causal relationship. Generally, OCPC occurs in the period after an individual completes his/her EA; therefore, it is difficult to eliminate the influence of various confounding factors on the association between OCPC and education. In contrast, studies have shown that EA is a genetically traceable phenotype and that MR can reveal the causal effect of EA on complex diseases (27). In this study, MR was used to clarify the relationship between EA and OCPC. The results show that an increase in educational level was significantly associated with a decline in the risk of OCPC (including OPC and OCC), supporting the evidence from the above mentioned observational clinical studies.

Additionally, we also explored the mediators in the EA–OCPC pathway. The preliminary results showed that BMI, hypertension, type 2 diabetes, and smoking may play important roles in this pathway. A previous MR study evaluated the impact of smoking on OCPC risk and indicated that smoking significantly increased OCPC risk (28). To correct for the above mediating factors, the MVMR model was further applied. However, MVMR analysis showed that EA was no longer significantly related to these factors, except for alcohol consumption. Therefore, these factors seem unlikely to play a role in the EA–OCPC path. It must be pointed out that our results showed that higher education may increase the risk of alcohol consumption, which was consistent with the results of previous research (29). Of note, even after excluding intermediary factors, higher education still had a protective role against OCPC.

However, the mechanism by which education affects OCPC remains unclear. Several studies have posited the following potential explanations. People with higher education may have a healthier lifestyle and access to better healthcare (30, 31) and may be more likely to encounter and understand the relevant information on OCPC than those with low levels of EA, subsequently avoiding “risk-taking” behaviors (26). People with higher education live on campus for a longer time than people with lower education, which is conducive to forming a healthier lifestyle (32). Individuals who attain high levels of education are less likely to start smoking, reduce the amount of smoking, and increase the likelihood of quitting smoking (33). Previous studies have confirmed that smoking plays a causal role in OCPC (34). Moreover, higher education can reduce the risk of HPV infection (10), which is a potential risk factor for OPC, and improve the rate of HPV vaccination.

The current meta-analysis had several limitations. First, the analysis incorporated case-control studies, and questionnaires or interviews were used to collect information in most studies. Second, this meta-analysis had some heterogeneity, which may be related to the following factors: different methods for measuring education level, potential confounding factors, different data sources, and inclusion of participants from all over the world. Finally, our analyses were not adjusted for age, sex, smoking, or alcohol abuse.

There were also several limitations to the current MR analysis. First, the data used for analysis could not be stratified by covariates such as age, sex, smoking, and alcohol abuse. Second, the study population used to determine the exposure and outcome was of European descent, which may have reduced the bias caused by population stratification. However, it is unclear whether these findings can be extrapolated to populations of other nationalities. Third, the selected EA-related dataset of 766,345 people included 442,183 participants from the UK Biobank. Therefore, we must recognize that the overlapping of participants between exposure and outcome may lead to substantial bias (35). Finally, MR results may only partially explain the causal effect of EA on OCPC because genetic variation may not accurately reflect the level of education in reality, and the occurrence of OCPC is determined by genetic and environmental factors.

## 5. Conclusions

This meta-analysis and MR study provided robust evidence on the effect of EA on the risk of OCPC. Therefore, reducing the prevalence of OCPC in individuals with low educational levels to ensure early detection and treatment should be the focus of public health policies. Moreover, we urgently need to expand the level of education and implement science/education programs for promoting healthy behaviors, especially in educationally disadvantaged areas.

## Data availability statement

The original contributions presented in the study are included in the article/Supplementary material, further inquiries can be directed to the corresponding authors.

## Author contributions

GC, JX, XZ, and AT: conceptualization and design. XZ and AT: project administration. GC, DL, and JX: data analysis and interpretation. All authors: manuscript writing and final approval of manuscript.
