# Supplementary figures and images for: Causal effects of education attainment on oral and oropharyngeal cancer: New evidence from a meta-analysis and Mendelian randomization study
Source: Front Public Health. 2023 Apr 12;11:1132035. doi: 10.3389/fpubh.2023.1132035 (PMC10130402; doi:10.3389/fpubh.2023.1132035)

Funnel plot with pseudo 95% confidence limits

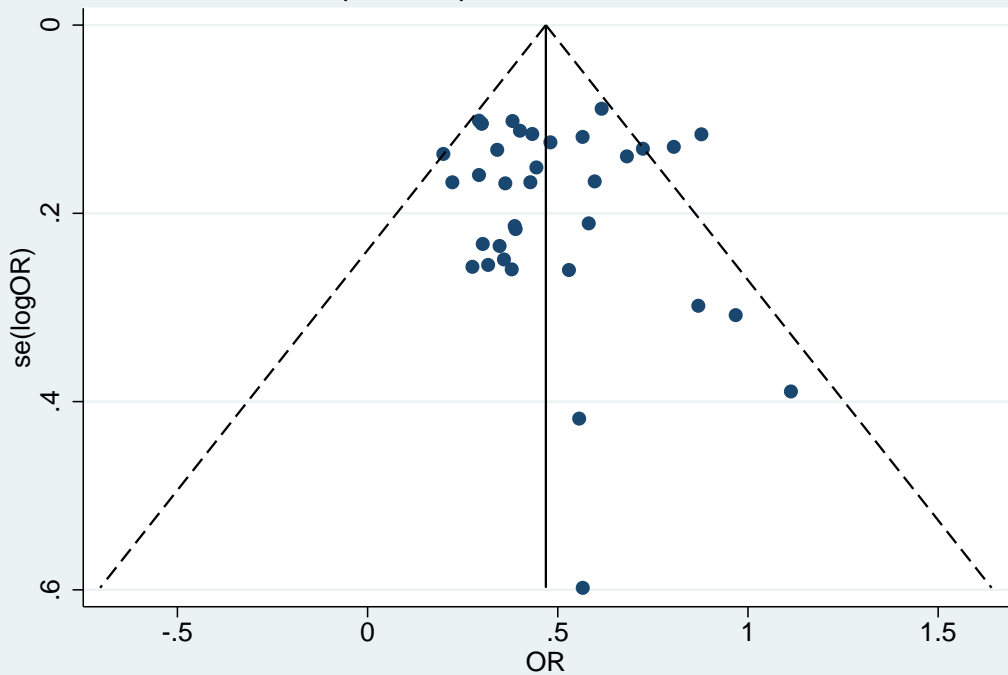

Supplement: Supplementary file 1 [file Data_Sheet_1.PDF]

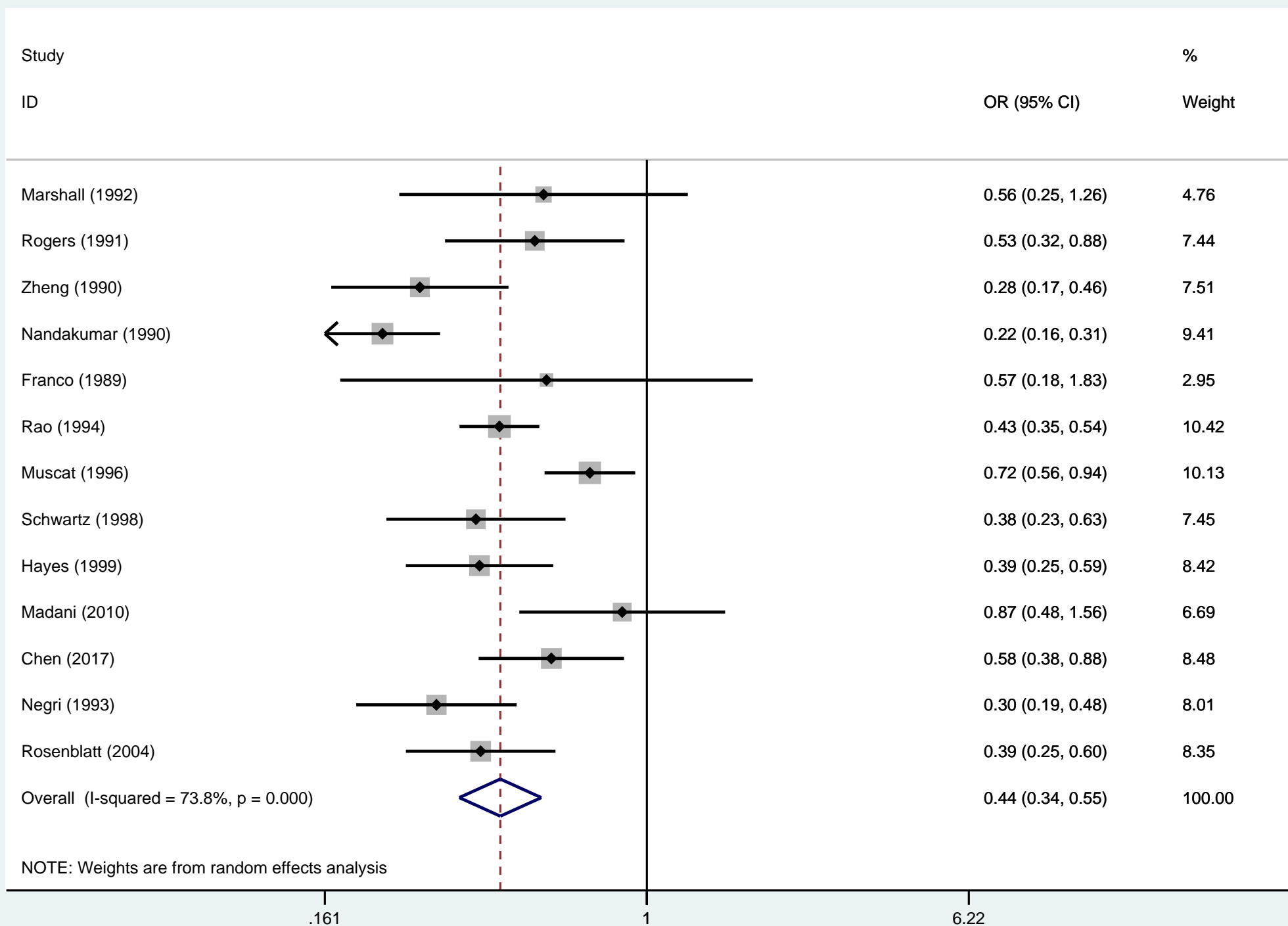

Supplement: Supplementary file 2 [file Data_Sheet_2.PDF]

MR Method

- Inverse variance weighted
- MR Egger

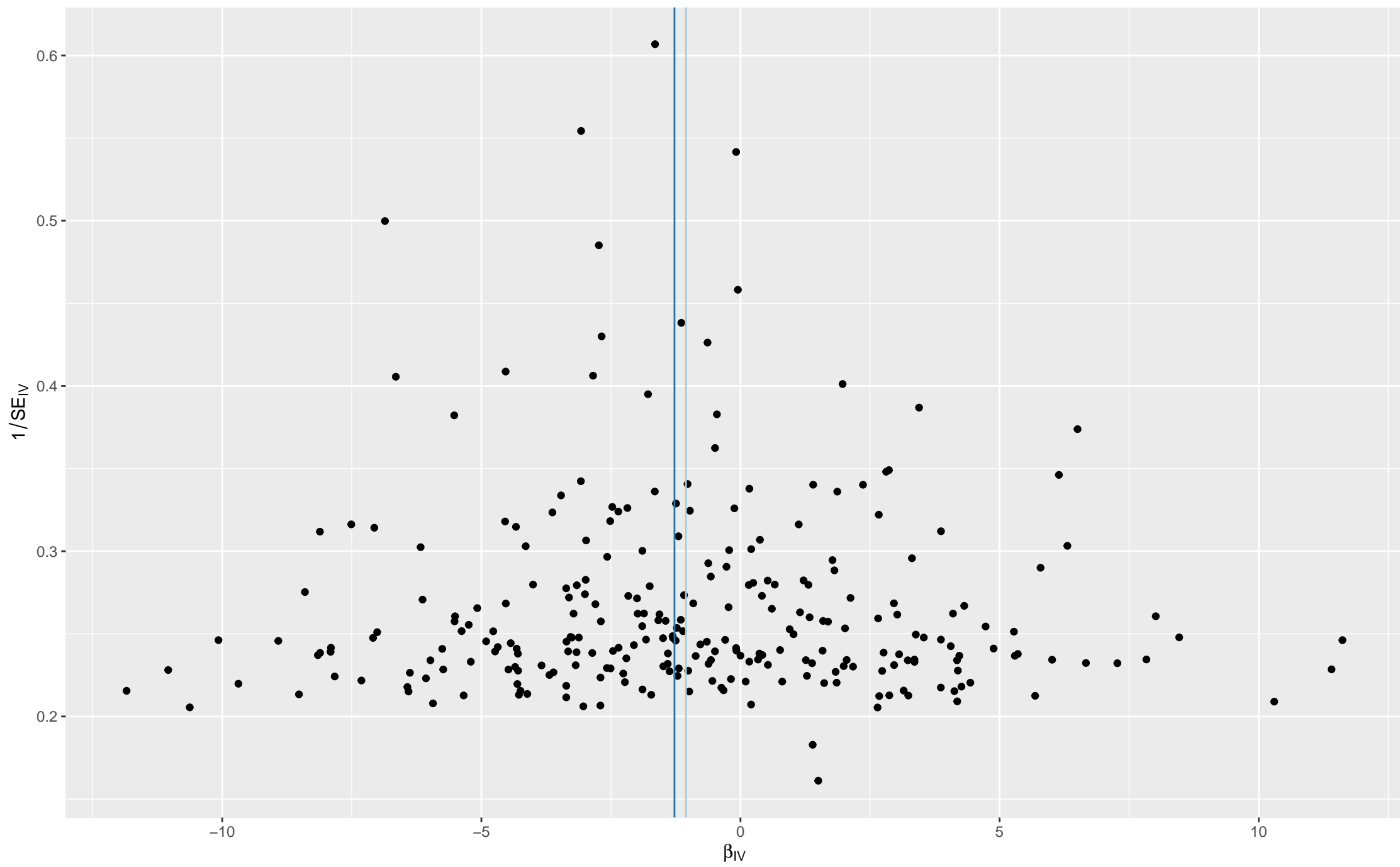

Supplement: Supplementary file 4 [file Data_Sheet_4.PDF]

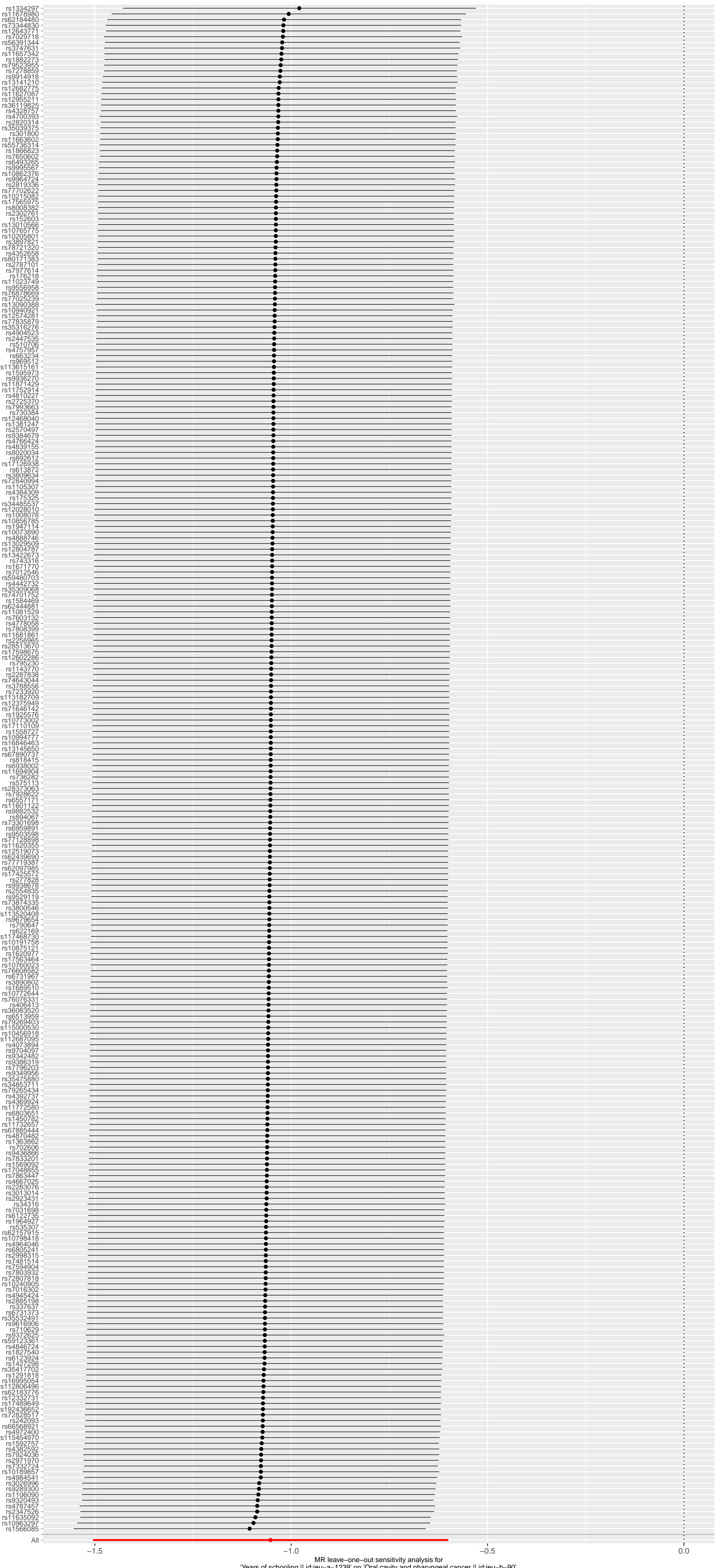

Supplement: Supplementary file 5 [file Data_Sheet_5.PDF]

A

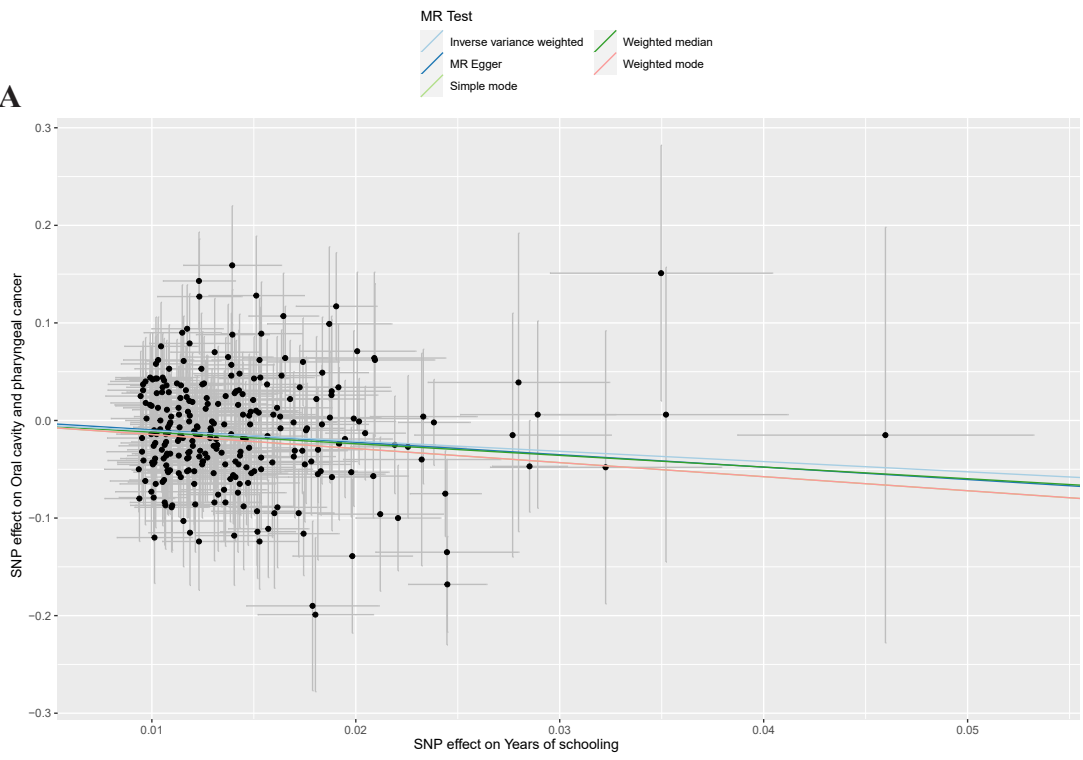

B

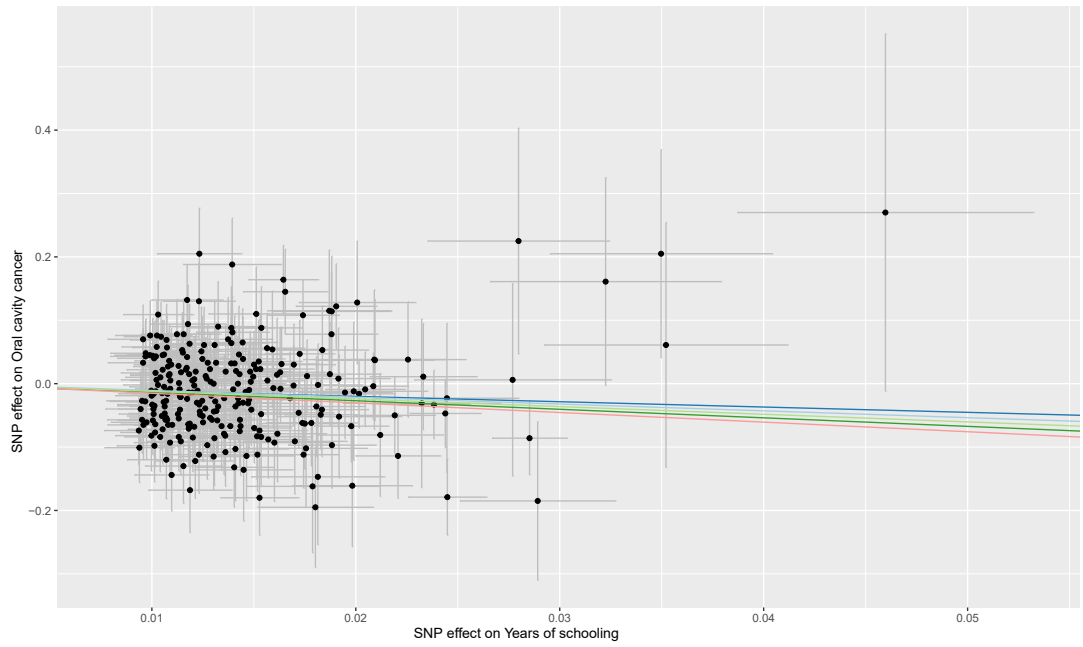

C

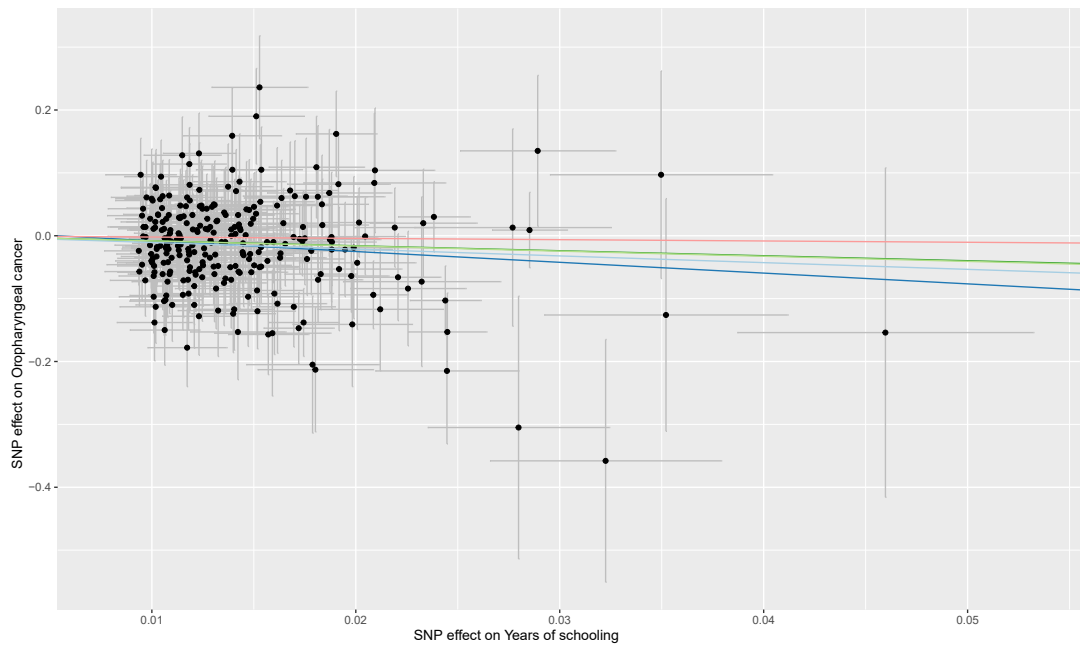

Supplement: Supplementary file 6 [file Data_Sheet_6.PDF]
