# Supplementary material for: Causal effects of education attainment on oral and oropharyngeal cancer: New evidence from a meta-analysis and Mendelian randomization study
Source: Front Public Health. 2023 Apr 12;11:1132035. doi: 10.3389/fpubh.2023.1132035 (PMC10130402; doi:10.3389/fpubh.2023.1132035)
Supplement: Supplementary file 3 [file Data_Sheet_3.PDF]

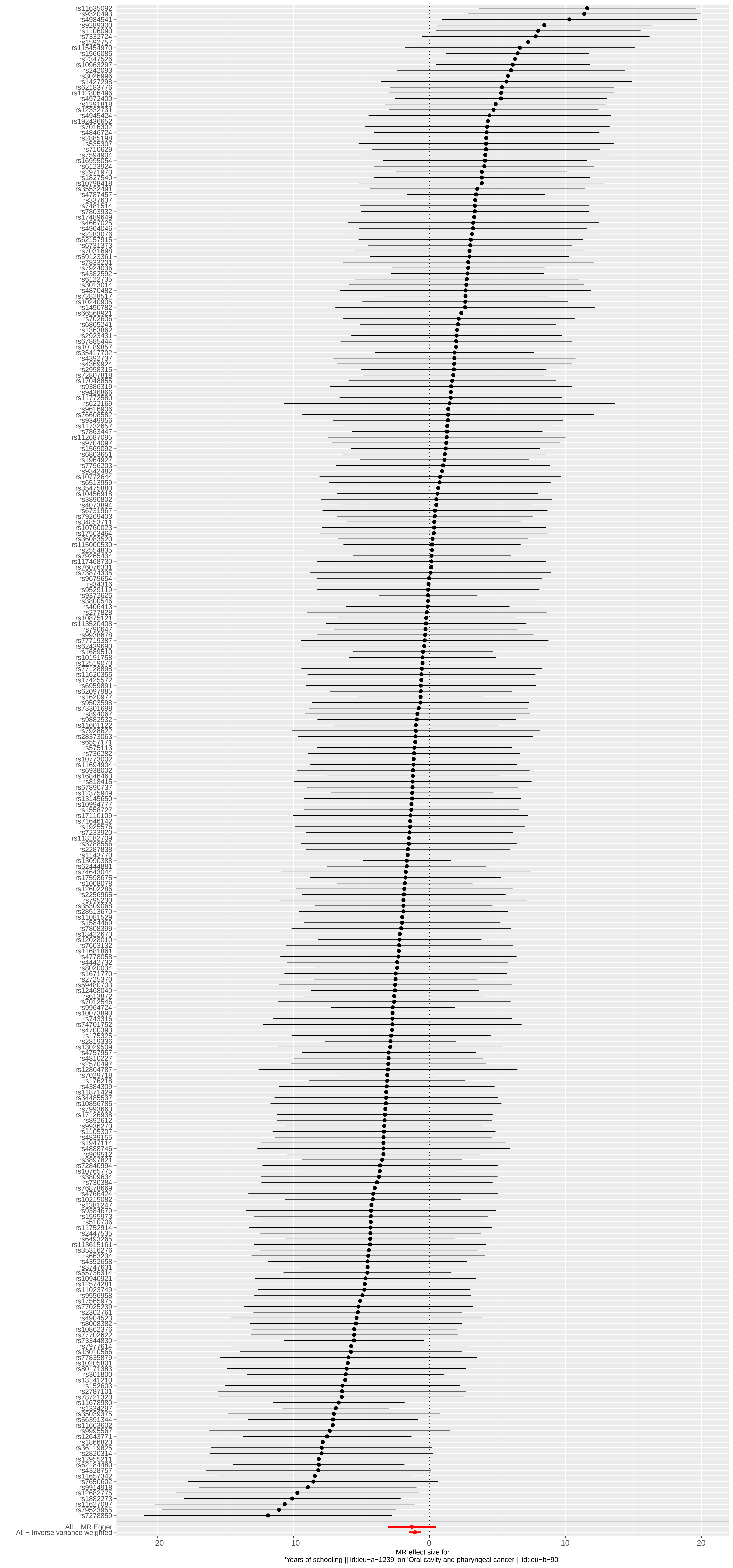

All − MR Egger
All − Inverse variance weighted
−20
−10
0
10
20
MR effect size for
'Years of schooling || id:ieu−a−1239' on 'Oral cavity and pharyngeal cancer || id:ieu−b−90'
